# Final-State Condition and Dissipative Quantum Mechanics

**DOI:** 10.3390/e24101411

**Published:** 2022-10-02

**Authors:** Pei-Ming Ho

**Affiliations:** Department of Physics and Center for Theoretical Physics, National Taiwan University, Taipei 106, Taiwan; pmho@phys.ntu.edu.tw

**Keywords:** black hole, Hawking radiation, information loss paradox

## Abstract

Unitarity demands that the black-hole final state (what remains inside the event horizon at complete evaporation) must be unique. Assuming a UV theory with infinitely many fields, we propose that the uniqueness of the final state can be achieved via a mechanism analogous to the quantum-mechanical description of dissipation.

## 1. Introduction

Recently, there has been great interest in the information loss paradox of black holes. A recent development that has attracted a lot of attention is a new way to calculate the entropy of Hawking radiation [1,2], with results consistent with the Page curve [3,4]. On the other hand, the physical mechanism behind the entropy formula remains mysterious, although it must be some quantum gravity effect (e.g., microscopic wormholes in the ER = EPR proposal [5]). As an effort to understand the underlying physics in further detail, in this work, we study a stronger condition of unitarity.

Horowitz and Maldacena proposed [6] that the unitarity of black-hole evaporation can be preserved by imposing a unique final state as a boundary condition at the singularity of the gravitational collapse. It was then challenged [7] that this condition may not be sufficient to ensure unitarity. Nevertheless, we now show in this section that a necessary condition of unitarity is the uniqueness of the final state of the black hole.

**Figure 1:**
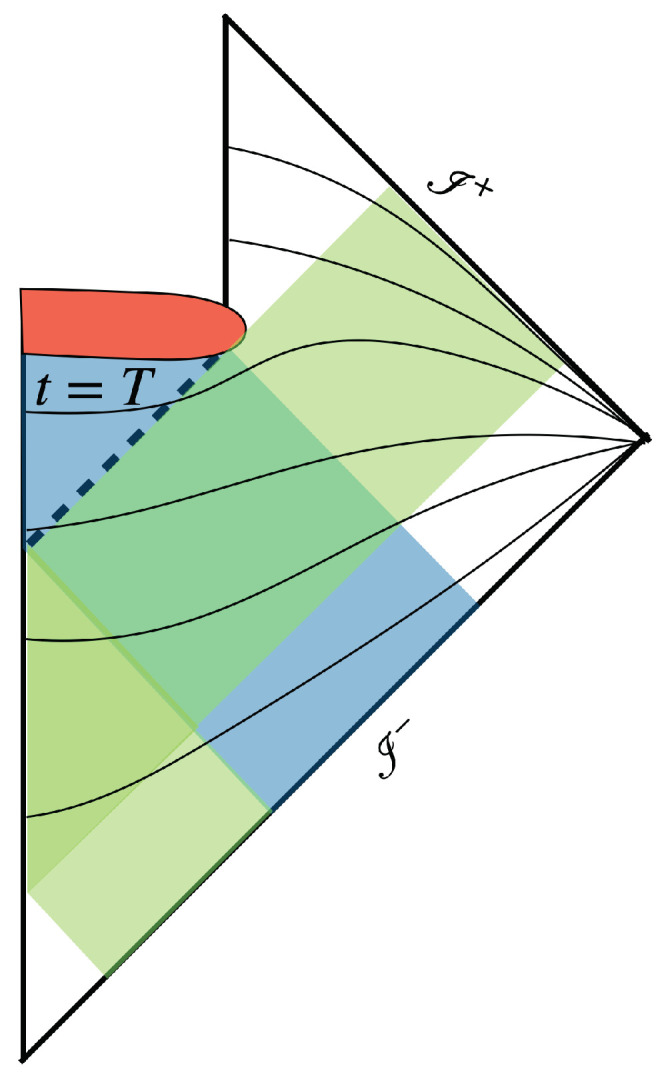
Penrose diagram of a black hole. The Hilbert space $H_{in}$ describes ingoing modes (the blue region), including the collapsing matter, that end up at the singularity (red blob). Outgoing modes in the green region end up outside the event horizon (dash line) belong to $H_{out}$. The ingoing and outgoing modes intersect outside the event horizon. The infinite past and infinite future are denoted as $I^{-}$ and $I^{+}$, respectively. Smooth time slices are schematically represented by the solid curves. The time slice just before the singularity at t=T is labelled.

The Hilbert space of a black hole is composed of $H_{in}$ for the ingoing quantum modes that eventually end up at the spatial singularity (a neighborhood of Planckian physics in the UV theory), and $H_{out}$ for the outgoing modes that eventually come out of the horizon, including Hawking radiation. (See Figure 1)— The Hilbert space for ingoing (outgoing) modes that never enter (exit) the horizon is irrelevant. For simplicity, we assume spherical symmetry and consider only scalar fields and Schwarzschild black holes in this work.

To deduce a necessary condition for unitarity, we first consider the special case of a pure initial state $|\Phi_{0}\rangle =|\phi_{0}\rangle ⊗|\Psi_{0}\rangle \in H_{in}⊗H_{out}$. Assuming unitarity, it remains a pure state under time evolution:

(1) $$\begin{array}{c} {|\Phi }_{0}\rangle \to |\Phi^{\prime }\rangle =\sum_{n}|\phi_{n}^{\prime }\rangle ⊗|\Psi_{n}^{\prime }\rangle , \end{array}$$

where $|\phi_{n}^{\prime }\rangle \in H_{in}$ and $|\Psi_{n}^{\prime }\rangle \in H_{out}$. At the end of the assumed complete evaporation, the space inside the horizon disappears from the viewpoint of external observers; hence, unitarity demands that the projection of $|\Phi^{\prime }\rangle$ onto $H_{out}$ is a pure state by itself. That is, the final state must be of the form |Φ〉=|ϕ〉⊗|Ψ〉.

If, for a set of different initial states $|\Phi_{0}^{\left(n\right)}\rangle =|\phi_{0}^{\left(n\right)}\rangle ⊗|\Psi_{0}\rangle$, where $|\Psi_{0}\rangle$ is the Minkowski vacuum of the infinite past, the corresponding final states are $|\Phi^{\left(n\right)}\rangle =|\phi^{\left(n\right)}\rangle ⊗|\Psi^{\left(n\right)}\rangle$, their superposition gives the time evolution

(2) $$\begin{array}{c} |\Phi_{0}\rangle =\left(\sum_{n}c_{n}|\phi_{0}^{\left(n\right)}\rangle \right)⊗|\Psi_{0}\rangle \;\to \;|\Phi \rangle =\sum_{n}c_{n}|\phi^{\left(n\right)}\rangle ⊗|\Psi^{\left(n\right)}\rangle . \end{array}$$

The entanglement entropy of the black hole would not vanish (that is, the final state $|\Phi^{\prime }\rangle$ is not a pure state for external observers) unless $|\phi^{\left(n\right)}\rangle =|\phi^{\left(0\right)}\rangle$ is a *unique* state for all *n* at complete evaporation. We will refer to this requirement as the *final-state condition*.

Notice that, even when the initial state of the outgoing modes is not exactly the Minkowski vacuum $|\Psi_{0}\rangle$, a unique final state $|\phi^{\left(0\right)}\rangle$ for the ingoing modes is still needed for unitarity. Hence, the uniqueness of the final state for the ingoing modes must be suitably robust against variations in the initial state. That is, for the class of initial states $|\phi_{0}^{\left(n\right)}\rangle ⊗|\Psi_{0}^{\left(n\right)}\rangle$ which admit complete evaporation, the final state of the ingoing modes should be exactly the same $|\phi^{\left(0\right)}\rangle$.

More generally, in a practical setting, the ingoing modes and outgoing modes are entangled in the initial state described by a density matrix

(3) $$\begin{array}{c} \rho_{0}=\sum_{m,n}\rho_{mn}|\phi_{0}^{\left(m\right)}\rangle ⊗|\Psi_{0}^{\left(m\right)}\rangle \langle \phi_{0}^{\left(n\right)}|⊗\langle \Psi_{0}^{\left(n\right)}|. \end{array}$$

At the end of the complete evaporation, the space inside the horizon disappears, and the density matrix ρ is effectively reduced to its trace $tr_{H_{in}}\left(\rho \right)$ over the Hilbert space of the ingoing modes. For a generic density matrix ρ, taking the trace over a subspace of the Hilbert space removes a lot of the information in ρ. However, the uniqueness of the final state described above demands that it evolves to a density matrix of the form

(4) $$\begin{array}{c} \rho =\sum_{m,n}\rho_{mn}|\phi^{\left(0\right)}\rangle ⊗|\Psi^{\left(m\right)}\rangle \langle \phi^{\left(0\right)}|⊗\langle \Psi^{\left(n\right)}|. \end{array}$$

In this case, taking the trace over $H_{in}$ amounts to merely removing the unique factor $|\phi^{\left(0\right)}\rangle \langle \phi^{\left(0\right)}|$ from ρ:

(5) $$\begin{array}{c} Tr_{H_{in}}\left(\rho \right)=\sum_{m,n}\rho_{mn}|\Psi^{\left(m\right)}\rangle \langle \Psi^{\left(n\right)}|, \end{array}$$

without removing any information in ρ. As the unitarity condition for mixed states is the same for pure states, we will restrict our discussions below to pure states for simplicity.

In a consistent quantum theory of gravity that respects unitarity and resolves singularities, the uniqueness of the final state should be achieved as a result of time evolution, instead of being imposed as a boundary condition. Thus, a natural question arises: what kind of mechanisms can bring an arbitrary initial state $|\phi_{0}^{\left(n\right)}\rangle$ to the same final state $|\phi^{\left(0\right)}\rangle$? We propose that the answer to this question is closely related to the familiar phenomena of dissipation.

## 2. Dissipative Quantum Mechanics

A ubiquitous feature of the macroscopic world is dissipation. A macroscopic oscillator *q* is better described by the equation

(6) $$\ddot{q}+\gamma \dot{q}+\omega^{2}q=0$$

with a constant γ>0 parametrizing the damping effect. Regardless of the initial state, eventually, q→0 as t→∞. As the underlying quantum system is unitary, the information about the initial state cannot be lost. How is the information preserved in the quantum system? The answer to this information loss problem is well known [8,9,10,11,12,13,14].

As a simple example, we consider the coupling of a simple harmonic oscillator (q,p) with a system of practically infinitely many oscillators $\left\{q_{i},p_{i}\right\}$. The total Hilbert space is $H_{total}=H_{osc}⊗H_{S}$. In the Heisenberg picture, the quantum states are time independent, and the operators evolve with time. It was found that the solutions of q(t) and p(t) are given by [10,11,12]

(7) $$\begin{array}{c} q\left(t\right)=e^{-\gamma t/2}q_{osc}\left(t\right)+\tilde{q}\left(t\right),\;p\left(t\right)=e^{-\gamma t/2}p_{osc}\left(t\right)+\tilde{p}\left(t\right), \end{array}$$

where

(8) $$\begin{array}{c} q_{osc}\left(t\right)\equiv q\left(0\right)\cos\left(\omega^{\prime }t\right)+p\left(0\right)\sin\left(\omega^{\prime }t\right),\;p_{osc}\left(t\right)\equiv p\left(0\right)\cos\left(\omega^{\prime }t\right)-q\left(0\right)\sin\left(\omega^{\prime }t\right), \end{array}$$

are the solutions of an undamped oscillator with a shifted frequency $\omega^{\prime }$, and $\tilde{q}\left(t\right)$ and $\tilde{p}\left(t\right)$ are operators on $H_{S}$. The damping parameter γ depends on the coupling between the oscillator (q,p) and the large quantum system $\left\{q_{i},p_{i}\right\}$. According to Equations (7) and (8), $\tilde{q}\left(0\right)=\tilde{p}\left(0\right)=0$, and q(0) and p(0) act only on $H_{osc}$.

For a given quantum state $|\Phi \rangle \equiv |\phi \rangle ⊗|\Psi \rangle \in H_{osc}⊗H_{S}$, where |ϕ〉 is normalized, the solution (7) implies that

(9) $$\begin{array}{c} \langle \Phi |q^{m}\left(t\right)p^{n}\left(t\right)|\Phi \rangle \to \langle \Psi |{\tilde{q}}^{m}\left(\infty \right){\tilde{p}}^{n}\left(\infty \right)|\Psi \rangle \;\left(t\to \infty \right). \end{array}$$

Hence, the information of the initial state in $H_{osc}$ is no longer accessible through the observables q(t) and p(t) in the limit t→∞.

In the Schrödinger picture, the operators are constant, and the quantum state evolves with time. Equation (9) means that the initial state |ϕ(0)〉 evolves into a state that is independent of |ϕ(0)〉, but dependent on |Ψ(0)〉.

At the same time, the unitarity of quantum mechanics ensures that all the information of the initial state |ϕ(0)〉 is preserved in the final state |Ψ(∞)〉 of other oscillation modes. This point is less emphasized in the literature on dissipative quantum mechanics. We demonstrate it via a toy model in the Appendix A.

## 3. Unique Final State

There is no mechanism in low-energy effective theories to transfer the complete information inside the collapsing matter into Hawking radiation. UV physics must become relevant, one way or another. (It has been advocated that, for the sake of unitarity, there must be O(1)-correction at the horizon [15], e.g., a firewall [16], which invalidates the effective theory. For concrete models, see, e.g., the fuzzball proposal [17,18,19] and the KMY model [20,21,22,23,24,25,26,27]. The discussion here is independent of the details of the model). Indeed, it was recently shown [28,29] that, as a manifestation of the trans-Planckian problem [30] of Hawking radiation, low-energy effective theories break down due to generic higher-derivative interactions.

In this work, we simply assume that the UV physics is at work between the ingoing modes (including the collapsing matter) and the outgoing modes in a certain neighborhood of the black hole. Furthermore, we assume that there is an infinite spectrum of fields in the UV theory. Apart from the well-known example of string theory, the existence of an infinite number of fields appears to be a salient feature of UV-finite, unitary quantum field theories that admit a perturbative formulation [31,32,33,34,35,36,37].

Note that, in a perturbative UV theory, even with the assumption of trans-Planckian scatterings, it is still unclear how the final-state condition can be satisfied. In the previous section, we pointed out a mechanism that brings an oscillator to “forget” about its initial state. We now translate this mechanism to a UV theory with infinitely many fields.

If we focus on a small neighborhood around a given point in spacetime, each quantum mode is associated with a pair of conjugate operators that can be identified with those of an oscillator. For each ingoing mode of an arbitrary field, we identify its creation and annihilation operators with (q,p) in the previous section, up to a linear transformation. The operators $\left\{q_{i},p_{i}\right\}$ are then identified with the outgoing modes of all other fields.

The initial state of the ingoing modes is $|\phi \left(0\right)\rangle \in H_{osc}$, on which (q,p) acts. The initial state $|\Psi \left(0\right)\rangle \in H_{S}$ is the Unruh vacuum (which is the time evolution of the Minkowski vacuum of the infinite past).

The discussion in the previous section implies that the initial state |ϕ(0)〉⊗|Ψ(0)〉 of the black hole evolves towards a state |ϕ(∞)〉⊗|Ψ(∞)〉 with |ϕ(∞)〉 independent of |ϕ(0)〉. The final state |ϕ(∞)〉 is unique. The final-state problem would be solved, (see the Appendix A for a toy model) except that the spacetime geometry is cut off by the space-like singularity at the origin. As we are not sure how the singularity would be resolved in the UV theory, we restrict ourselves to t≤T, with t=T beginning at a time slice just before the singularity emerges. Here, we assume that the time coordinate *t* is globally defined, as time slices defined by equal *t* are schematically depicted in Figure 1.

The characteristic time scale $\gamma^{-1}$ of this dissipative mechanism is typically of the same order of magnitude as the characteristic length scale of the UV theory (quantum gravity) which is the Planck length $\ell_{p}$. As long as the time span of the region where UV physics is at work is much longer than $\ell_{p}$, in the Heisenberg picture, Equation (7) implies that

(10) $$\begin{array}{c} q\left(T\right)≃\tilde{q}\left(T\right),\;p\left(T\right)≃\tilde{p}\left(T\right). \end{array}$$

The same argument applies to all ingoing modes. We can talk about all the ingoing modes at the same time. The tensor product of $H_{osc}$ for all ingoing modes is $H_{in}$, which is defined in Section 1, and $H_{S}$ can be identified with $H_{out}$. Let $P_{in}$ and $P_{out}$ denote the projections onto the Hilbert spaces $H_{in}$ and $H_{out}$, respectively. Equation (10) means that, in the Schrödinger picture, the state $P_{in}|\phi \left(T\right)\rangle$ of the ingoing modes is approximately independent of |ϕ(0)〉. As it can only depend on |Ψ(0)〉, which is unique, the final state $P_{in}|\phi \left(T\right)\rangle$ is approximately unique. (The information of the initial state |ϕ(0)〉 is preserved in the state $P_{S}|\Psi \left(T\right)\rangle$ of the outgoing modes).

Notice that, as we have defined the final state at a time *T* before the singularity emerges, the singularity at r=0 is not directly related to the final-state problem. Furthermore, all we need is an approximate uniqueness of the final state, as the singularity should be resolved in the UV theory as a Planckian neighborhood, which may still carry some information.

Apart from the question of whether we need it to solve the final-state problem, dissipation is a robust feature of any system with infinite degrees of freedom. The evidence is the simple fact that dissipation (e.g., friction) appears everywhere. It is sufficient to have a UV region much larger than the Planck length, where UV–theory interactions have to be treated as a continuous effect, in contrast with brief interactions in scattering processes.

## 4. Comments

We answer some of the natural questions here.

*How is this work different from the proposal of Ref.* [6] *?*

Our proposal is closely related to, but quite different from the proposal of the final-state boundary condition [6]. We propose here that the final state of the collapsing matter is unique as the result of a robust feature of UV theories with infinitely many fields (while a spectrum of infinitely many fields is argued to be a common feature of perturbative formulation of UV theories). The information is transferred through causal, local interactions. However, it is necessary to assume that low-energy effective theory breaks down, and UV physics is important somewhere other than the singularity.

On the other hand, in the proposal of Horowitz and Maldacena, the final-state boundary condition is imposed by hand at the space-like singularity of a Schwarzschild black hole, presumably as an effective description of certain unknown UV physics. As opposed to our proposal, the infalling negative-energy Hawking radiation behind the horizon plays the crucial role of a medium for the post-selected teleportation of quantum information in their theory, and everywhere other than the singularity at the origin can be smooth and uneventful. However, in this scenario, causality is at risk [7,38], and it is not clear whether it can be realized by a certain UV mechanism.

*What do we really mean by $H_{in}$?*

In our statement of the final-state condition, we have implicitly assumed that the Hilbert space can be decomposed into two parts: $H_{in}$ is the Hilbert space for everything that ends up inside the event horizon, and $H_{out}$ the Hilbert space for everything that ends up outside the event horizon. Strictly speaking, the existence of the event horizon is uncertain, as the singularity at the center of the black hole is expected to be resolved in the UV theory. Nevertheless, when the black hole evaporates to 1/n of its initial mass, the entanglement entropy of the state in $H_{in}$ should be ∼O(2/n) for a large *n* according to the Page curve [3]. This means that the part of the quantum state in $H_{in}$ deviates from the unique state only by a tiny fraction. Lastly, the approximate uniqueness of the final state has to be explained.

*What about other types of black holes?*

In this work, we have focused on the Schwarzschild black hole, i.e., spherically symmetric neutral black holes in asymptotically flat spacetime. For charged black holes, the final states are extremal black holes with given charges. As they do not completely evaporate, the final state inside the horizon does not necessarily have to be unique. Nevertheless, before the radiation stops, it is possible that part of the information carried by the collapsing matter is transferred into Hawking radiation through the dissipation process in UV physics.

There are also black holes without spherical symmetry, e.g., those with angular momentum, and those in spacetimes with different asymptotic properties, e.g., AdS black holes. As a ubiquitous phenomenon, dissipation persists whenever there is Hawking radiation, regardless of whether it leads to a unique final state. We do not yet have a detailed understanding of the effect of dissipation in these cases. This work is merely the first step in understanding the role played by dissipation in UV physics.

*Do we really need infinitely many fields?*

In the analogue between quantum mechanics (QM) and quantum field theories (QFT), a single quantum field is equivalent to infinitely many quantum mechanical degrees of freedom. Does this mean that a single field can do the job of the infinitely many particles in dissipative quantum mechanics? In the perturbative formulation of QFT, a particle in an asymptotic state moves freely until it is spatially close to another particle. There is no continuous interaction with other degrees of freedom, as depicted in the dissipation process, unless it passes through a high density of particles. This is why we do not see the dissipative effect at low energies.

In our scenario, dissipation only takes place in a finite region of spacetime outside the horizon. (In Figure 1, dissipation occurs in the finite region where the blue and green stripes overlap.) In principle, this region can be very small, (e.g., Planckian scale) so we need a very efficient dissipation for an approximately unique final state. For the toy model in the Appendix A, infinite time is needed for a complete information transfer through dissipation with a single field. Hence, we need a large number of fields to achieve the needed efficiency.

*Does the same mechanism apply to different models of black holes?*

For the spacetime geometry of the conventional model of black holes, the evaporation time scale $O(a^{3}/\ell_{p}^{2})$ for a distant observer is causally matched with the proper time scale $O(\ell_{p})$ from the viewpoint of a freely falling observer comoving with the collapsing matter [39,40]. (Here, *a* is the Schwarzschild radius and $\ell_{p}$ the Planck length). For the KMY model, the collapsing matter’s proper time scale of evaporation is O(a). It is still a very short time scale to burn out all the collapsing matter unless we have interactions at the Planck scale. The mechanism outlined above provides such a violent mechanism, which is applicable to both models.

In the conventional model of black holes, the collapsing matter passes through the horizon without “dramma”. It is still a mystery how a UV theory is relevant in view of the decoupling principle. This is a key problem in the information loss paradox to which this paper has not provided any answers. Instead, we comment on the KMY model [20,21,22,23,24,25,26,27]. In the KMY model, UV physics is needed at the outer layers of the collapsing matter, due to a Planckian pressure associated with outgoing radiation. Matter evaporates layer by layer from the outside. Nevertheless, how matter is completely turned into radiation cannot be fully explained in the low-energy effective theory. Our proposal provides a UV mechanism behind this process, and tells us that it only takes Δt∼ several $\ell_{p}$ to incinerate each layer.

## 5. Conclusions

In the sections above, we have proposed that the same mathematics of dissipative quantum mechanics provide a mechanism for UV theories with infinitely many fields to satisfy the final-state condition and to transfer the information of the collapsing matter to outgoing radiation. The mechanism behind this proposal is robust, and it is applicable to almost all models of black-hole evaporation as long as the UV theory of quantum gravity consists of an infinite number of fields.

To conclude, we have shown that the quantum mechanical origin of the classical phenomenon of dissipation helps resolve the final-state problem of black holes. It also explains how the information of the collapsing matter is simultaneously transferred into outgoing UV modes.

## Data Availability

Not applicable.

## Appendix A. Toy Model

In this appendix, we construct a toy model of dissipative quantum mechanics suitable for application to black holes. The same qualitative features are argued in the literature to persist in more general models [8,9,10,11,12,13,14].

Consider the coupling of a harmonic oscillator (q,p) with a system of infinitely many oscillators ${\left\{q_{n},p_{n}\right\}}_{n\in Z}$ with the following Lagrangian

$$\begin{array}{c} L=\frac{1}{2}m{\dot{q}}^{2}\left(t\right)-\frac{1}{2}m\omega^{2}q^{2}\left(t\right)+\lambda \left(t\right)q\left(t\right){\dot{q}}_{0}\left(t\right)+\sum_{n\in Z}\left(\frac{1}{2}{\dot{q}}_{n}^{2}\left(t\right)-\frac{1}{2}M^{2}q_{n}^{2}\left(t\right)+\tilde{\lambda }q_{n}\left(t\right)q_{n+1}\left(t\right)\right). \end{array}$$

Without loss of generality, we set m=1. For simplicity in the calculation below, we choose $M^{2}=2\tilde{\lambda }>0$. In the continuum limit

(A1) $$\begin{array}{c} n\to {\tilde{\lambda }}^{1/2}\sigma ,\;q_{n}\to {\tilde{\lambda }}^{-1/4}\psi \left(t,\sigma \right), \end{array}$$

the Lagrangian becomes

(A2) $$\begin{array}{c} L=\frac{1}{2}{\dot{q}}^{2}\left(t\right)-\frac{1}{2}\omega^{2}q^{2}\left(t\right)+\lambda \left(t\right)q\left(t\right)\dot{\psi }\left(t,0\right)+\int_{-\infty }^{\infty }d\sigma \;\left(\frac{1}{2}{\dot{\psi }}^{2}\left(t,\sigma \right)-\frac{1}{2}{\psi^{\prime }}^{2}\left(t,\sigma \right)\right). \end{array}$$

We will now analyze the dissipation mechanism of this model.

The Euler–Lagrange equations are

(A3) $$\begin{array}{cc} \ddot{q}\left(t\right)+\omega^{2}q\left(t\right)-\lambda \left(t\right)\dot{\psi }\left(t,0\right) & =0, \end{array}$$

(A4) $$\begin{array}{cc} \ddot{\psi }\left(t,\sigma \right)-\psi^{\prime \prime }\left(t,\sigma \right)+\frac{d}{dt}\left(\lambda (t)q(t)\right)\delta \left(\sigma \right) & =0. \end{array}$$

The general solution of Equation (A4) is

(A5) $$\begin{array}{c} \psi \left(t,\sigma \right)=\psi_{h}\left(t,\sigma \right)-\frac{1}{2}\lambda \left(t-|\sigma |\right)q\left(t-|\sigma |\right), \end{array}$$

where the homogeneous solution $\psi_{h}\left(t,\sigma \right)\equiv \psi_{+}\left(t+\sigma \right)+\psi_{-}\left(t-\sigma \right)$ for arbitrary functions $\psi_{\pm }$. Plugging Equation (A5) into Equation (A3), we find

(A6) $$\begin{array}{c} \ddot{q}+\frac{1}{2}\lambda^{2}\left(t\right)\dot{q}\left(t\right)+{\hat{\omega }}^{2}\left(t\right)q\left(t\right)=\lambda \left(t\right){\dot{\psi }}_{h}\left(t,\sigma \right), \end{array}$$

where ${\hat{\omega }}^{2}\equiv \omega^{2}+\frac{\lambda \left(t\right)\dot{\lambda }\left(t\right)}{2}$. This is the equation of a damped oscillator. Its general solution is

(A7) $$\begin{array}{cc} q(t) & =q_{h}\left(t\right)+\tilde{q}\left(t\right). \end{array}$$

The homogeneous solution $q_{h}\left(t\right)$ describes an oscillation with an exponentially decaying amplitude. The special solution is

(A8) $$\begin{array}{cc} \tilde{q}\left(t\right) & \equiv \int_{-\infty }^{\infty }dt^{\prime }\;K\left(t,t^{\prime }\right)\lambda \left(t^{\prime }\right){\dot{\psi }}_{h}\left(t^{\prime },0\right), \end{array}$$

where the retarded Green’s function is

(A9) $$\begin{array}{c} K\left(t,t^{\prime }\right)\equiv \Theta \left(t-t^{\prime }\right)\;e^{-\int_{t^{\prime }}^{t}\frac{\lambda^{2}\left(t^{\prime \prime }\right)}{4}dt^{\prime \prime }}\;\frac{\sin(\omega \left(t-t^{\prime }\right))}{\omega }, \end{array}$$

where Θ is the step function. The derivation of the solution for p(t) is straightforward.

When we apply this toy model to the evaporating black hole, we identify (q,p) with any infalling propagating quantum mode, and identify $\left\{q_{n},p_{n}\right\}$ (or $\left\{\psi ,\dot{\psi }\right\}$) with the outgoing propagating modes of another field. Around a given time during the evaporation of the black hole, the UV theory is needed only in a certain neighborhood of spacetime. The coupling λ(t) in the UV theory is thus turned on only when the ingoing and outgoing fields intersect in this neighborhood. Without loss of generality, we turn on the coupling λ(t) at t=0. That is,

(A10) $$\begin{array}{c} \lambda \left(t\right)=\lambda_{0}\Theta \left(t\right). \end{array}$$

Strictly speaking, the interaction is turned off after a certain critical time $T^{\prime }$ so we should have written $\lambda \left(t\right)=\lambda_{0}\left(\Theta \left(t\right)-\Theta \left(t-T^{\prime }\right)\right)$. We have chosen Equation (A10) because our purpose here is to observe the exponential decay of an oscillation mode due to dissipation. Furthermore, since the singularity inside the horizon is expected to be removed by UV physics at the Planckian scale; we only need to explain the uniqueness of the final state as an approximate description. Hence, Equation (A10) is suitable for our purpose as long as $T^{\prime }$ is sufficiently large.

The solutions of *q* and ψ are now given by

(A11) $$\begin{array}{cc} q(t) & =q_{h}\left(t\right)+\lambda_{0}\int_{0}^{t}dt^{\prime }\;e^{-\frac{\lambda_{0}^{2}\left(t-t^{\prime }\right)}{4}}\frac{\sin(\omega \left(t-t^{\prime }\right))}{\omega }{\dot{\psi }}_{h}\left(t^{\prime },0\right), \end{array}$$

(A12) $$\begin{array}{cc} \psi (t,\sigma ) & =\psi_{h}\left(t,\sigma \right)-\frac{1}{2}\lambda_{0}\Theta \left(t-|\sigma |\right)q\left(t-|\sigma |\right), \end{array}$$

where $q_{h}\left(t\right)$ is given by Equation (7) with

(A13) $$\begin{array}{c} \gamma =\frac{\lambda_{0}^{2}}{2},\;\omega^{\prime }=\sqrt{\omega^{2}-\frac{\lambda_{0}^{4}}{16}}. \end{array}$$

In the Heisenberg picture, the state $|\phi \rangle ⊗|\Psi \rangle \in H_{osc}⊗H_{S}$ of the quantum system is constant and the operators evolve with time. As $q=q_{h}$ and $\psi =\psi_{h}$ at the initial time t=0, the Hilbert spaces $H_{osc}$ and $H_{S}$ are representations of $\left(q_{h},{\dot{q}}_{h}\right)$ and $\left(\psi_{h},{\dot{\psi }}_{h}\right)$, respectively. At large *t* ($t\gg 1/\lambda_{0}^{2}$), q(t) becomes independent of $q_{h}$; hence, it can no longer extract information of the state $|\phi \rangle \in H_{osc}$. Instead, q(t) becomes dominated by the contribution of $\psi_{h}$ at large *t*. In the Schrödinger picture, this means that the state |ϕ(t)〉 of the collapsing matter becomes independent of its own initial state |ϕ(0)〉 at large *t*, but it would be determined by the initial state $|\Psi \left(0\right)\rangle \in H_{S}$ of the outgoing UV fields. As the initial state |Ψ(0)〉 is fixed (the Unruh vacuum), the final state |ϕ(∞)〉 is unique.

Another conclusion we draw from the general solution (A11), (A12) is the following. In the Heisenberg picture, although the state |ϕ〉 becomes inaccessible to the operators (q(t),p(t)) at large *t*, it is still accessible at any time *t* through the operators ψ(t,σ=±t) and $\dot{\psi }\left(t,\sigma =\pm t\right)$, which include $q_{h}$ and ${\dot{q}}_{h}$ without the exponential suppression factor $e^{-\lambda_{0}^{2}t/4}$. In the Schrödinger picture, this means that the information of the initial state |ϕ(0)〉 is preserved in the state $|\Psi \left(t\right)\rangle \in H_{S}$ at large *t*.
